# Mental health problems and influencing factors in Japanese women 4 months after delivery

**DOI:** 10.1186/1880-6805-33-32

**Published:** 2014-10-29

**Authors:** Naoko Yamamoto, Yasuyo Abe, Kazuhiko Arima, Takayuki Nishimura, Emi Akahoshi, Kazuyo Oishi, Kiyoshi Aoyagi

**Affiliations:** Department of Public Health, Nagasaki University Graduate School of Biomedical Sciences, 1-12-4, Sakamoto, Nagasaki, 852-8523 Japan; Department of Reproductive Health, Unit of Nursing, Nagasaki University Graduate School of Biomedical Sciences, 1-7-1, Sakamoto, Nagasaki, 852-8102 Japan

**Keywords:** Depression, Financial difficulty, GHQ-12, Mental health, Postpartum, Regular eating

## Abstract

**Background:**

Postpartum mental health problems are a major public health issue; however, studies on the mental health status of mothers and its influencing factors between 8 weeks and 1 year postpartum are scarce. Furthermore, it would be necessary to examine the factors influencing mothers’ mental health in order to evaluate their physiological adaptations to the nursing environment.

**Methods:**

We examined the mental health status of postpartum women and the factors influencing poor mental health at 4 months after delivery. A cross-sectional study of 584 postpartum women was conducted. Information on mental health status, delivery, and other factors was collected using a self-administered questionnaire. Women were asked about their age, height, weight, gestational or marital status, whether they were eating regular meals, appetite, frequency of going out, financial difficulty, stressful life events, and history of depression. The Japanese version of the 12-item General Health Questionnaire (GHQ-12) was used to identify potential poor mental health status. Participants with GHQ-12 scores of ≥4 were classified as the high GHQ-12 score group (poor mental health status) and participants with GHQ-12 scores of ≤3 were classified as the low GHQ-12 score group (good mental health status).

**Results:**

Forty-five women (7.7%) were classified as having high GHQ-12 scores. Multiple logistic regression analysis revealed that older age, not eating meals regularly, and history of depression were significantly associated with poor mental health. Financial difficulty had a borderline association with poor mental health in this model.

**Conclusions:**

These risk factors might help practitioners identify women at high risk of poor mental health after delivery.

## Background

Postpartum mental health problems are a major public health issue [1]. The most common of these problems is postpartum depression, which usually occurs 4 to 6 weeks after childbirth [2] but can occur up to 1 year postpartum [3]. The prevalence of postpartum depression has been reported to be 10 to 14% [4–7]. Postpartum depression can have adverse effects not only on affected mothers, but also on their children’s development and on the well-being of the whole family [8–10].

The 12-item General Health Questionnaire (GHQ-12) is a valid, internationally used measure of mental health status in a population. High GHQ-12 scores have frequently been used to identify the possible presence of psychological disorders. Several studies used the GHQ-12 in order to assess postpartum mothers’ mental health status [11–13].

Some of the reported risk factors of postpartum depression are low social status, stressful life events, psychological disturbance during pregnancy, a poor marital relationship, low social support, and past history of psychopathology [7, 14, 15]. Studies on the mental health status of mothers have mostly been conducted 4 to 8 weeks [12, 16, 17] or 1 year [11, 18, 19] after delivery. However, it is critical to gain better insight into mothers’ mental health between 8 weeks and 1 year postpartum to fully understand postpartum mental health problems. Furthermore, it would be necessary to examine the factors influencing mothers’ mental health in order to evaluate their physiological adaptations to the nursing environment. Therefore, the purpose of this study was to assess the mental health condition of mothers 4 months after delivery and to elucidate the factors influencing poor mental health.

## Participants and methods

A cross-sectional study was conducted in two healthcare centers in Nagasaki, Japan, from November 2011 to July 2012. A total of 646 women whose infants underwent a 4-month health examination at these centers were invited to participate in this study, and 602 women gave written informed consent to participate (participation rate: 93.2%). This study was approved by the Ethical Committee of Nagasaki University, Graduate School of Biomedical Sciences (approval number 11090863).

### Questionnaire

Information on mental health status, delivery, and other factors was collected using a self-administered questionnaire. Women were asked about their age, height, weight, gestational age at delivery, parity (primiparous/multiparous), method of delivery (vaginal/caesarean section), gestational stage at delivery (preterm, full term, or post term), marital status (married or living with a partner, single, divorced, or widowed), whether the pregnancy was planned (yes/no), method of infant feeding (breastfeeding, partially breastfeeding, or formula feeding), whether they were eating meals regularly (yes/no), appetite (good/poor), frequency of going out (almost every day, 2 to 3 times a week, once a week, 1 to 2 times a month, or less than once a month), financial difficulty (yes/no), stressful life events (yes/no), and history of depression (yes/no).

The Japanese version of the GHQ-12 was used to identify potential poor mental health status. High GHQ-12 scores have frequently been used to identify the possible presence of psychological disorders. The 12 items assessed by the GHQ-12 are lost sleep, feelings of being under strain, inability to concentrate, feeling unable to play a useful role, inability to face problems, inability to make decisions, inability to overcome difficulties, feeling unhappy, lack of enjoyment in day-to-day activities, feeling depressed, lack of confidence, and feelings of worthlessness. Responses were given on a 4-point scale (corresponding to the presence of symptoms: “not at all,” “same as usual,” “slightly more than usual,” or “much more than usual”), and were scored in a bimodal fashion (0-0-1-1). Total scores ranged from 0 to 12, with higher scores indicating that the respondent experienced more symptoms of psychological distress. Participants with GHQ-12 scores of ≥4 were classified as the high GHQ-12 score group and participants with GHQ-12 scores of ≤3 were classified as the low GHQ-12 score group.

### Statistical analysis

Participants with any missing questionnaire responses were excluded, leaving 584 women for the final analysis. The χ^2^ test, Fisher exact test, and Student’s *t-*test were used to assess differences between the high and low GHQ-12 score groups. Multiple logistic regression analysis was used to evaluate the simultaneous effects of variables on high GHQ-12 scores. Odds ratios and 95% confidence intervals were calculated. Starting with a full model including all variables, the most appropriate model was selected based on Akaike’s information criteria. *P* values <0.05 were considered significant. Statistical analysis was performed using SPSS software version 19 for Windows (SPSS Japan, Tokyo, Japan).

## Results

Table 1 shows the characteristics of the participants. The mean (standard deviation) age of participants was 31.4 (5.1) years old. The majority (86.8%) of the women ate meals regularly, 20% reported financial difficulty, and 6.2% reported a history of depression.

**Table 1:** **Participant characteristics**

		(n =584)
Variable		
	Average	SD
Age (years)	31.4	5.1
Height (cm)	158.1	5.3
Weight (kg)	51.9	7.0
Gestational age at delivery (weeks)	38.9	1.5
	**n**	**%**
Parity		
Primiparous	279	47.8
Multiparous	305	52.2
Methods of birth		
Vaginal birth	507	86.8
Caesarean section	77	13.2
Term		
Preterm (<37 weeks)	30	5.1
Full term (37–41 weeks)	547	93.7
Post term (≥42 weeks)	7	1.2
Marital status		
Married or living with partner	567	97.1
Single	13	2.2
Divorced	4	0.7
Widowed	0	0
Pregnancy		
Planed	440	75.3
Not planed	144	24.7
Breastfeeding status		
Fully breastfeeding	383	65.6
Partially breastfeeding	143	24.5
Formula feeding	58	9.9
Eating meals regularly		
Yes	507	86.8
No	77	13.2
Appetite		
Good	559	95.7
Poor	25	4.3
Frequency of going out		
Almost everyday	255	43.7
2–3 times a week	251	43.0
Once a week	61	10.4
1–2 times a month	13	2.2
Less	4	0.7
Financial difficulty		
No	467	80.0
Yes	117	20.0
Stressful life events experienced		
Yes	183	31.3
No	401	68.7
History of depression		
Yes	36	6.2
No	548	93.8

Table 2 shows the univariate comparison of variables between the high and low GHQ-12 score groups. Forty-five women (7.7%) were classified as having high GHQ-12 scores (poor mental health status), and their mean age was significantly older compared to women with low GHQ-12 scores (good mental health status). Women with high GHQ-12 scores showed significantly higher prevalence of not eating meals regularly, poor appetite, financial difficulty, and history of depression compared to women with low GHQ-12 scores.

**Table 2:** **Comparison of the high (poor mental health status) and low (good mental health status) GHQ-12 score groups**

						(n =584)
Variable		High GHQ-12 score		Low GHQ-12 score		***P***value
		(n =45)		(n =539)		
		Average	SD	Average	SD	
Age (years)		33.6	5.5	31.2	5.1	<0.01
Height (cm)		158.3	5.1	158.1	5.3	0.80
Weight (kg)		51.7	7.3	51.9	6.9	0.86
Gestational age at delivery (weeks)		39.0	1.6	38.9	1.5	0.86
		N	%	N	%	
Parity	Primiparous	22	48.9	257	47.7	0.50
Method of birth	Caesarean section	4	8.9	73	13.5	0.49
Term	Preterm	4	8.9	26	4.8	0.28
Marital status	Single or Divorced	3	6.3	14	2.6	1.00
Pregnancy	Not planned	16	35.6	128	23.7	0.10
Breastfeeding status	Partially breastfeeding or Formula feeding	21	46.7	180	33.4	0.08
Eating meals regularly	No	12	26.7	65	12.1	0.01
Appetite	Poor	5	11.1	20	3.7	0.04
Frequency of going out	No or 1–2 times a month	1	5.9	16	94.1	1.00
Financial difficulty	Yes	16	35.6	101	18.7	0.01
Stressful life events experienced	Yes	14	31.1	169	31.4	1.00
History of depression	Yes	11	24.4	25	4.6	0.01

Multiple logistic regression analysis revealed that older age, not eating meals regularly, and history of depression were significantly associated with high GHQ-12 score (Table 3). Financial difficulty had a borderline association with high GHQ-12 scores in this model. We added the analysis in women without history of depression. Older age and not eating meals regularly were significantly associated (data not shown).

**Table 3:** **Logistic regression analysis of factors associated with high GHQ-12 score**

Variable	Comparison	Odds ratio	95% Cl	***P***value
Age	One year increase	1.1	1.1–1.2	<0.01
Eating meals regularly	No/Yes	3.0	1.4–6.5	0.01
Financial difficulty	Yes/No	2.0	1.0–4.0	0.06
History of depression	Yes/No	6.0	2.6–14.0	<0.01

## Discussion

In the present study, 7.7% of mothers had poor mental health (high GHQ-12 score) at 4 months postpartum, with the factors associated with poor mental health status being older age, not eating meals regularly, history of depression, and financial difficulty.

The prevalence of poor mental health (GHQ-12 score of ≥4) among women 4 months after delivery observed in this study was lower than previous reports on postpartum mothers [12, 13]. In Italy, the prevalence of poor mental health among women 6 to 8 weeks after delivery was 13% [12]. Furthermore, a study in the United Kingdom reported that 23.5% of women 3 to 6 months after delivery had a GHQ-12 score of ≥4 [13]. These differences may be due to the cultural background and environment specific to each country. A study comparing the rates of psychological distress of women 1 year after childbirth in France, Italy, and Canada showed that differences between the rates in these countries were present, and that although the reason for the differences was unclear, distress may be expressed differently between countries [11]; therefore, careful attention is needed when interpreting results from different countries.

Nakao et al. [20] recently assessed the mental health status of the general population in Nagasaki, and reported that the prevalence of poor mental health (GHQ-12 score of ≥4) was 34.4% in women aged 20 to 39 years, and was 17.9% in women aged 40 to 64 years, which were both higher than the prevalence observed in our study. To date, there is no clear evidence of whether mental health problems are more or less common among postpartum women than non-postpartum women. One study reported higher prevalence of depressive disorder among postpartum women than a control group [21], another reported lower prevalence of depressive disorder among postpartum women than non-postpartum women [22], and others reported no significant difference between postpartum women and controls [13, 23]. Further studies are necessary to clarify this issue; however, considering the significant negative impact on mothers and children [8–10], postpartum mental health problems are definitely one of the most important global public health issues.

In the present study, older age was significantly associated with poor mental health as assessed by the GHQ-12 among postpartum women. However, some studies reported that younger maternal age was associated with postpartum mental health problems [24, 25]. On the other hand, Bjerke et al. [26] reported that among Pakistani women, the probability of postpartum depression at 6 to 12 weeks after delivery was 4.6 times higher in women over 30 years of age compared to women under 30. Other studies have reported no significant difference in mean age between postpartum women with and without poor mental health [16, 27]. As mentioned above, these differences may be due to the specific cultural and social background of each country. Further studies are needed to clarify this association.

Financial difficulty or financial stress have been associated with depressive symptoms and mental health problems [28, 29]. A study on mental health at the population level in Finland showed that a poor economic situation was associated with poor mental health status as assessed by the GHQ-12 [30]. Furthermore, some studies have pointed out that financial difficulties are a significant risk factor for postpartum mental health problems [18, 24, 31]. Romito et al. [18] reported that financial worries were associated with poor mental health status in Italian and French women 12 months after birth. Ngoma et al. [24] also reported a significant association between financial difficulty and mental health problems among Japanese women 1 month postpartum and speculated that this association might be partly attributed to increased levels of stress among mothers who lack the financial means necessary to raise their infants. In our study, financial difficulty had a borderline association with poor mental health, which is consistent with previous findings. New mothers with financial difficulty might represent a group that should be targeted to prevent poor mental health.

We showed that history of depression was significantly associated with poor mental health among Japanese mothers 4 months postpartum. Panthangi et al. [32] used the Edinburgh Postpartum Depression Scale to assess postpartum depression in women who had given birth during the previous 5 to 8 weeks and showed the relationship between postpartum depression and history of depression. Saleh et al. [27] also reported that prior psychiatric problems were a significant predictor of postpartum depression as assessed by the Edinburgh Postpartum Depression Scale. Our study together with previous reports suggests that, for women with prior history of depression, appropriate screening and early intervention may be needed to prevent postpartum mental health problems.

A previous study showed that not eating meals regularly was associated with poor mental health status among community-dwelling Japanese women [20]. This finding was consistent with our results. To the best of our knowledge, there have been no previous studies on the relationship between the regularity of eating meals and mental health status among postpartum women. Eating patterns might vary considerably among different cultures and countries, which makes it somewhat difficult to evaluate on an international scale. However, irregular eating patterns may lead to mental health problems, and vice versa. Further study is required to fully understand this association among postpartum women.

Several limitations of the present study should be considered. Firstly, because this was a cross-sectional design, our results do not necessarily show a causal relationship. Longitudinal studies are required to establish relationships between poor mental health and the associated factors. Secondly, the present study included only Japanese women; therefore, it is not possible to extrapolate the results to women of other ethnicities. Thirdly, it was reported that depression patients have abnormalities in baseline cortisol secretion [33]. It is necessary to obtain data on cortisol levels in future research.

The prevalence of poor mental health was relatively low in women at 4 months postpartum in this study. Older age, not eating meals regularly, and a history of depression were associated with poor mental health. Financial difficulty had a borderline association with poor mental health. The risk factors identified in the present study might help practitioners identify women at high risk of developing poor mental health after delivery and aid in early intervention to prevent postpartum depression.

## Abbreviations

GHQ-12: 12-item General Health Questionnaire.
